# A rare complication of internal jugular venous catheterization in spinal fracture surgery: A case report

**DOI:** 10.1097/MD.0000000000046224

**Published:** 2025-12-12

**Authors:** Zhaolin Xie, Hongyuan Liao, Qibiao Zhang

**Affiliations:** a Department of Spine Surgery, Guigang People’s Hospital, Guigang, China.

**Keywords:** central venous catheterization, spinal fracture surgery

## Abstract

**Rationale:**

Central venous catheterization is common procedure in emergency and critical care settings to establish rapid vascular access. However, it carries a risk of severe complications, including catheter malposition.

**Patient concerns:**

A 56-year-old male patient presented with multiple fractures following severe trauma.

**Diagnoses:**

The patient was diagnosed with compression fractures of the T8 and L1 vertebrae with spinal canal stenosis. On postoperative day 3, routine chest imaging revealed malposition of the internal jugular vein catheter into the superior mediastinum, resulting in bilateral chylothorax.

**Interventions:**

The patient underwent posterior open reduction and pedicle screw fixation of the fractured vertebrae. Bilateral pleural drainage and supportive therapy were administered to manage the chylothorax.

**Outcomes:**

Following the interventions, the patient’s condition improved progressively, and he was discharged in stable condition on postoperative day 12.

**Lessons:**

This case highlights the importance of careful catheter placement and early recognition of rare complications such as catheter-related chylothorax to improve patient outcomes. Early recognition and prompt management are essential for preventing morbidity and improving patient outcomes.

## 1. Introduction

Central venous catheterization (CVC) involves the insertion of a specialized catheter into a central vein to establish rapid vascular access for fluid resuscitation and emergency treatment. It is extensively utilized in the management of critical conditions such as shock and acute circulatory failure.^[1]^ Although CVC is a common clinical procedure, the complications associated with this invasive procedure pose significant medical risks. Commonly reported complications include procedure-related issues such as arterial injury, pneumothorax, and chylothorax (common with left subclavian approach); catheter-related complications including malposition, dislodgement, obstruction, extravasation, thrombosis, infection, and catheter fracture; and other complications such as nerve injury, tachycardia, air embolism, cardiac perforation, cardiac tamponade, and even death.^[2]^ This article presents a rare case of right internal jugular vein catheter migration into the superior mediastinum resulting in bilateral pleural effusion during spinal fracture surgery.

## 2. Case report

A 56-year-old male patient was admitted on May 31, 2024, presenting with multiple body pain and limited mobility for about 3 hours following a fall from height. At approximately 9 am, the patient fell from a height of about 6 m, subsequently experiencing pain in his chest, abdomen, back, and lower extremities. He reported restricted movement in both lower limbs, accompanied by chest tightness and dyspnea. After initial treatment at a local clinic, he was transferred to our hospital via ambulance due to his critical condition. After emergency treatment including blood transfusion, intravenous fluid resuscitation, and anti-shock therapy, he was admitted with a preliminary diagnosis of severe multiple trauma and hemorrhagic shock.

Physical examination on admission revealed a clear level of consciousness, but the patient appeared acutely ill with notable pallor of the lips and mucous membranes. Pupils were 2.5 mm bilaterally, equal, and promptly reactive to light. The patient exhibited labored breathing with intercostal, suprasternal, and subcostal retractions. Pulmonary auscultation revealed coarse breath sounds bilaterally, with moist rales in the right lower lung field, and diminished breath sounds with moist rales in the left lung field. There was significatn tenderness to palpation and percussion over the thoracolumbar spine. Neurological examination showed muscle strength of grade 5/5 in the upper extremities and grade 4−/5 in the lower extremities, with normal muscle tone.

Vital signs revealed a body temperature of 36.8°C, a pulse rate of 99 beats/min, a respiratory rate of 23 breaths/min, and blood pressure (BP) of 79/54 mm Hg. Laboratory results indicated a white blood cell count of 13.5 × 10^9^/L, a hemoglobin level of 99 g/L, a myoglobin level of 226.64 ng/mL, and a creatine kinase-myocardial band level of 96.14 ng/mL.

**Figure 1.**
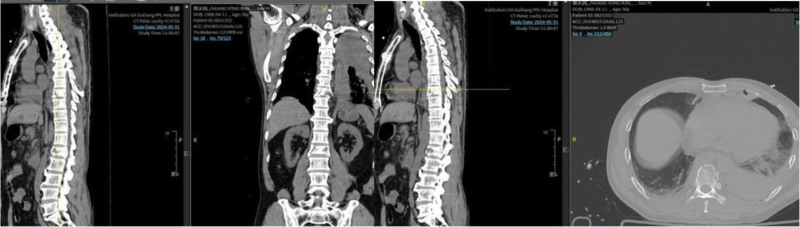
Computed tomography (CT) images of the thoracic and lumbar spine on admission. CT = computed tomography.

**Figure 2.**
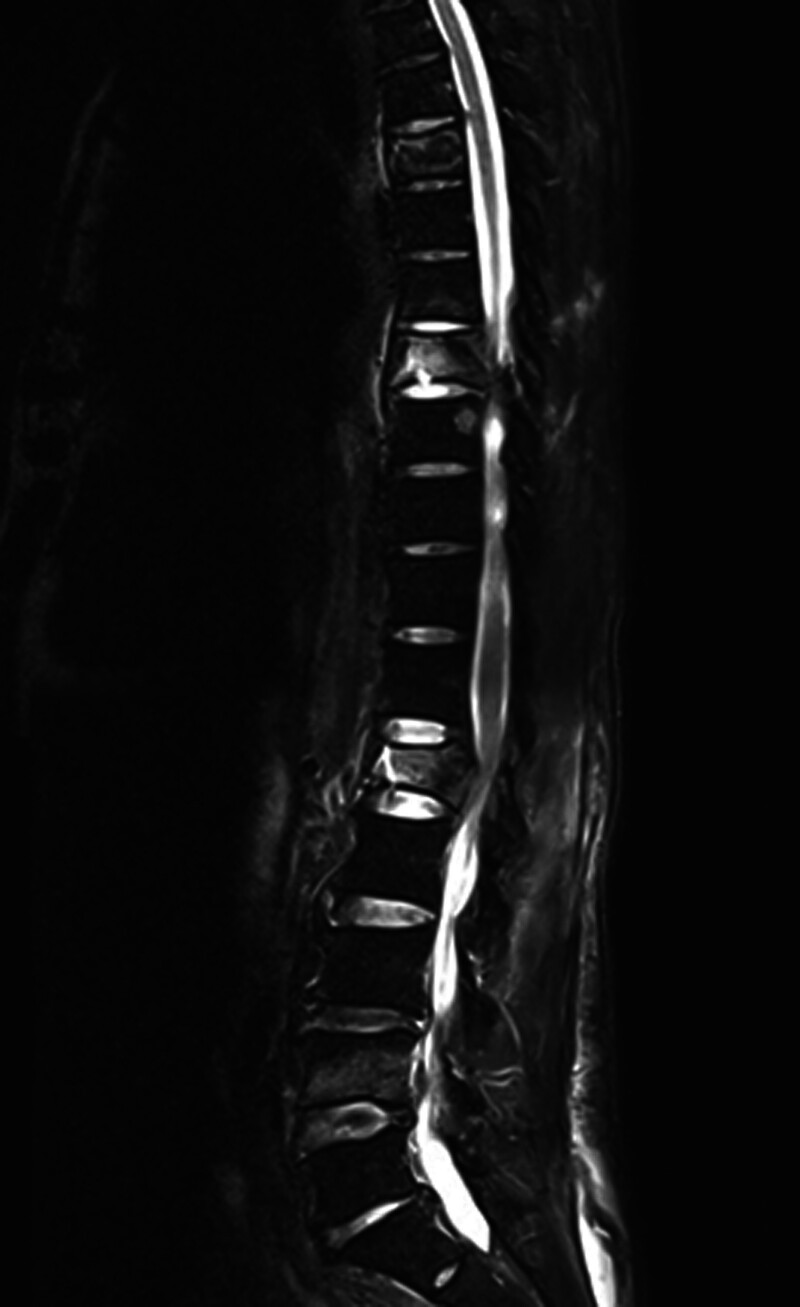
Sagittal T2-weighted magnetic resonance (MRI) images of the thoracic and lumbar spine. MRI = magnetic resonance imaging.

Imaging studies reveal multiple fractures. Chest computed tomography (CT) indicated bilateral pulmonary contusions and a left-sided hemopneumothorax with approximately 30% lung compression. Spinal CT revealed multiple fractures involving the T5, T7, T8, L1, L4, and S3 vertebrae, the first coccygeal vertebra, the L1 accessory processes, and the left transverse processes of T9, L2, and L3 (Fig. 1). Magnetic resonance imaging showed compression fractures with bone marrow edema at the T2, T5, T7, T8, L1, and L4 vertebrae, secondary spinal canal stenosis at L1 with spinal cord compression, bone marrow edema of the T7–T8 spinous process, and surrounding soft tissue swelling (Fig. 2).

The patient was diagnosed with multiple significant injuries, including T8 and L1 vertebral compression fractures with associated spinal canal stenosis; and incomplete paraplegia. Further diagnoses comprised a closed chest injury with fractures of the left 2nd to 5th costal cartilages, multiple fractures of the left 1st and 7th through 12th ribs, hemopneumothorax, and bilateral pulmonary contusion. Spinal imaging also revealed burst fractures of the T5, T7, L4, and S3 vertebrae, multiple fractures of the first coccygeal vertebra, L1 appendages, and left transverse processes of T9, L2, L3. Additionally, the patient presented with hemorrhagic shock.

After admission, the patient underwent comprehensive diagnostic evaluations, multidisciplinary consultations, and was managed with intensive care, mechanical ventilation, blood transfusion, anti-shock therapy, analgesia, and left chest closed thoracic drainage.

Following supportive treatment and stabilization of the patient’s general condition, posterior open reduction and pedicle screw internal fixation of T8 and L1 vertebral fracture was performed under general anesthesia on June 13, 2024. Upon arrival in the operating room, standard monitoring was initiated, and anesthesia was induced uneventfully, followed by successful endotracheal intubation. Anesthesia was maintained using a combination of intravenous and inhalational agents. After achieving stable anesthesia, right internal jugular vein catheterization was performed under strict aseptic technique with the patient in the Trendelenburg position. Venous access was successfully obtained, and a guidewire was smoothly advanced. Sequential dilation of the tissue tract was performed, followed by insertion of a double-lumen central venous catheter. The catheter was secured with sutures, demonstrated good blood return, and was connected to infusion lines for fluid and medication administration.

**Figure 3.**
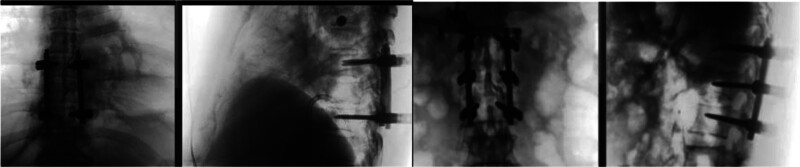
Intraoperative fluoroscopic images of the thoracic and thoracolumbar spine.

**Figure 4.**
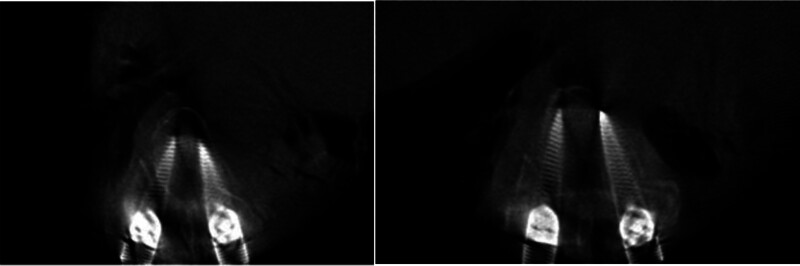
Intraoperative O-arm scanning of thoracic pedicle trajectories demonstrating optimal positioning of T7 and T9 screws. O-arm = O-shaped arm x-ray machine.

The patient was placed in the prone position, and O-shaped arm x-ray machine (O-arm) fluoroscopy was used for localization. After routine disinfection and draping, longitudinal incisions were made at T7–T9 and T12–L2 to expose the surgical field. Pedicle screws were inserted under O-arm navigation, and intraoperative fluoroscopy confirmed proper placement (Fig. 3). A window decompression was performed at L1 to relieve the dural sac, followed by deformity correction and rod fixation (Fig. 4). Intraoperative blood loss was approximately 300 mL. Given the patient’s multiple trauma and preoperative anemia, which posed a risk for postoperative respiratory and circulatory compromise, the patient was transferred to the intensive care unit for close monitoring and further management.

On postoperative day 1, the patient remained under continuous sedation and analgesia. Lumbar wound drainage was 100 mL, and left thoracic drainage was 50 mL. Vital signs were stable with a temperature of 37.1°C, pulse of 108 beats/min, respiratory rate of 21 breaths/min, BP of 115/66 mm Hg, oxygen saturation (SpO_2_) of 95% under ventilator support.

On postoperative day 2, sedation and analgesia were continued. Lumbar wound drainage was 120 mL, and left thoracic drainage increased significantly to 300 mL. Vital signs remained stable with a temperature of 36.8°C, pulse of 102 beats/min, respiratory rate of 21 breaths/min, BP of 117/67 mm Hg, and SpO_2_ of of 95% on mechanical ventilation.

**Figure 5.**
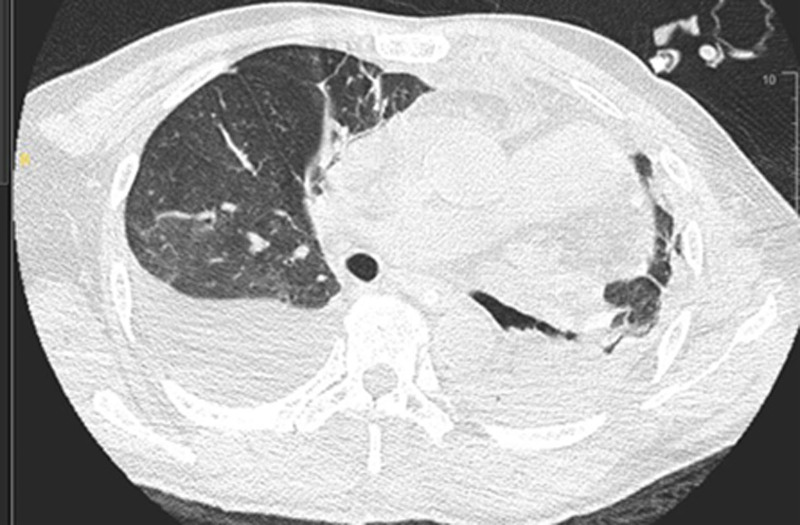
Postoperative day 3 computed tomography (CT) scan showing bilateral pleural effusions. CT = computed tomography.

On postoperative day 3, the left chest tube drained approximately 50 mL of milky, turbid fluid. An emergency chest CT revealed left lower lobe atelectasis, pulmonary consolidation, and a right pleural effusion. A right-sided tube thoracostomy was performed, draining 300 mL of milky fluid (Fig. 5).

Evaluation of the central venous catheter revealed brisk blood return from the side port, while the main port had no blood return, raising concern for potential catheter malposition or inadvertent venous perforation with subsequent placement in the thoracic cavity. As a precaution, infusions through the internal jugular central line was suspended, and consultations were initiated with the relevant specialties for further evaluation and management.

**Figure 6.**
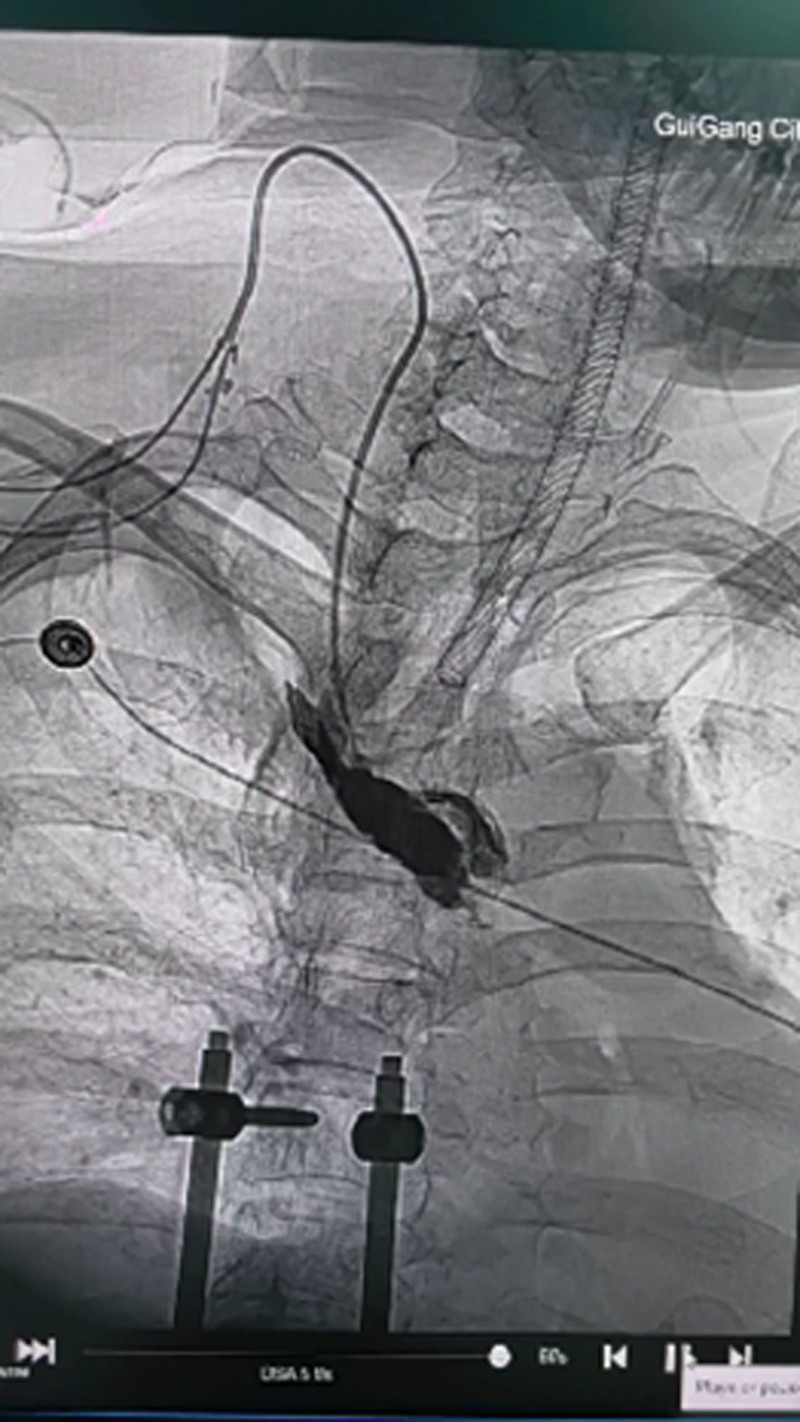
Right internal jugular venography on postoperative day 4.

**Figure 7.**
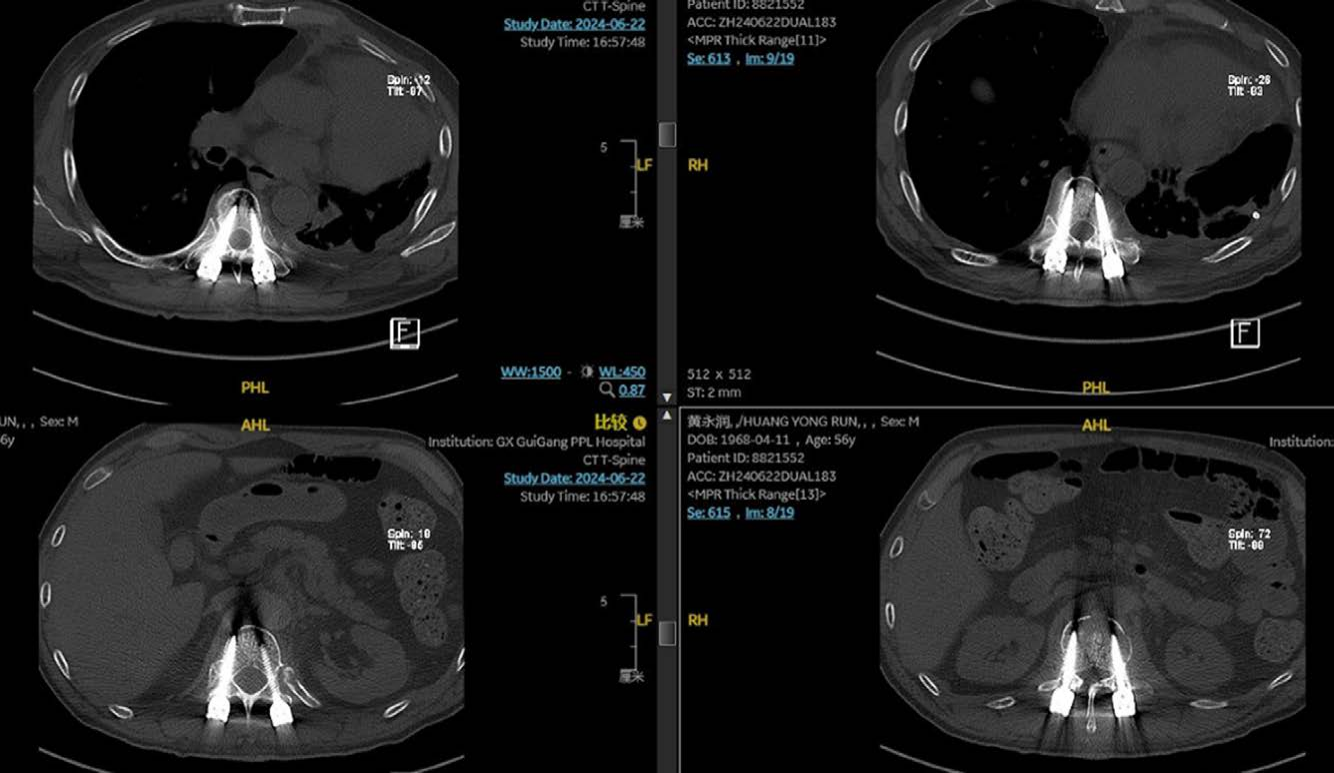
Thoracolumbar computed tomography (CT) on postoperative day 9. CT = computed tomography.

**Figure 8.**
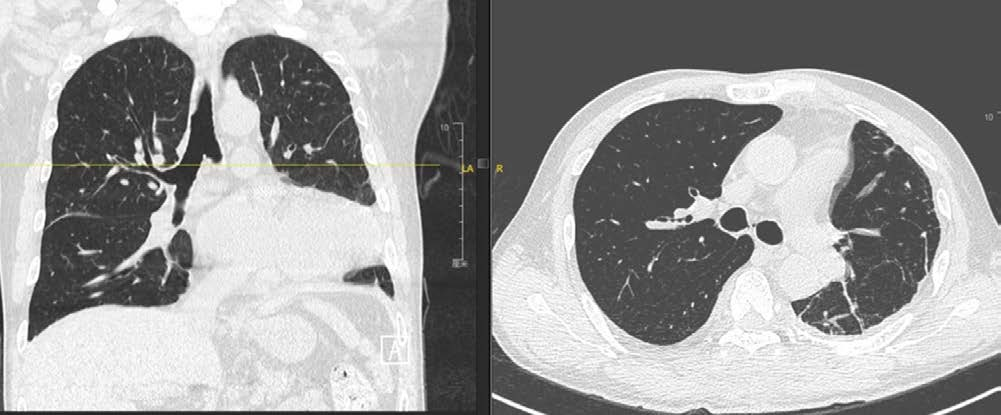
Postoperative chestcomputed tomography (CT) on day 10. CT = computed tomography.

To confirm the position of catheter, right internal jugular venography was performed. The study demonstrated contrast medium extravasation into the superior mediastinum, confirming vascular injury and intrathoracic penetration of the right internal jugular catheter (Fig. 6). Following discontinuation of infusions through the catheter, bilateral thoracic drainage volumes gradually decreased (Fig. 7). A follow-up chest CT performed on postoperative day 10 showed post-drainage changes in the left hemithorax without evidence of significant residual pleural effusion and the chest tube were subsequently removed (Fig. 8). The patient achieved full clinical recovery and was discharged on postoperative day 12.

Sagittal and coronal views show the overall alignment, and an axial view at T8 demonstrates the fracture morphology.

Venography performed via the right internal jugular vein on postoperative day 4 demonstrates contrast medium extravasation from the main lumen of the central venous catheter into the superior mediastinum, confirming vascular injury and catheter malposition.

Postoperative CT of the thoracolumbar spine obtained on day 9 demonstrates accurate pedicle screw placement and resolution of the right pleural effusion, confirming both effective spinal fixation and clinical improvement.

Axial chest CT obtained on postoperative day 10 shows near-complete resolution of bilateral pleural effusions, indicating effective thoracic drainage and recovery.

## 3. Discussion

CVC, as an invasive procedure, carries inherent risks. Complications, often related to technical difficulties or improper placement, include but are not limited to hemothorax, pneumothorax, arterial injury, catheter obstruction or dislodgement, and nerve injury.^[3]^ Nevertheless, this technique allows for long-term vascular access, thereby reducing the need for repeated venipunctures and minimizing patient discomfort.

In the present case, the central venous catheter tip inadvertently entered the thoracic cavity, a complication rarely documented in the medical literature. Su Hao’s team^[2]^ reported a similar case of subclavian venous catheterization entering the mediastinum. Their case involved a 38-year-old female who underwent subclavian vein cannulation for rectal cancer surgery. The patient developed chest tightness and dyspnea on postoperative day 2. An emergency bedside chest radiograph revealed bilateral lower lobe infiltrates and bilateral pleural effusions. Bilateral chest tube drainage was immediately performed, draining 900 and 800 mL of milky fluid from the left and right pleural spaces, respectively. On postoperative day 4, thoracic aorta computerized tomography angiography confirmed that the catheter had penetrated the venous wall and entered the superior mediastinum.

In our case, the patient underwent right internal jugular vein cannulation preoperatively, with smooth anesthesia and surgical procedure. By postoperative day 3, milky fluid was observed draining from both thoracic cavities. Blood could be aspirated through the central venous catheter in the neck, suggesting the catheter remained within the superior vena cava. Given the patient’s multiple thoracic vertebral fractures and recent posterior spinal instrumentation, the possibility of chylothorax secondary to spinal fracture or surgical pin placement could not be excluded.

Although rare, similar cases have been documented. For instance, Cui Ying’s team^[4]^ reported a case of a female patient admitted with chest and back pain for 6 days and chylous pleural effusion for 3 days following a traffic accident. Her diagnoses included: T11 vertebral fracture and traumatic chylothorax. The patient underwent open reduction and internal fixation of the T11 vertebral fracture, along with chest tube drainage, and conservative management. She was discharged 30 days post-injury with full resolution of the effusion.

Chylothorax results from disruption or obstruction of the thoracic duct, leading to accumulation of chyle within the pleural space. The thoracic duct, the body’s largest lymphatic vessel, measures approximately 30 to 40 cm in length. It originates from the cisterna chyli, located anterior to the first lumbar vertebra in the abdominal cavity, and ascends through the aortic hiatus of the diaphragm into the posterior mediastinum. It then courses along the anterior aspect of the thoracic vertebral bodies, posterior to the esophagus, crossing from right to left at approximately the level of the fifth thoracic vertebra. It continues cephalad along the left side of the vertebral column and esophagus, passing behind the carotid sheath and arching over the left subclavian artery before terminating at the left venous angle, the junction of the left jugular and left subclavian veins.

In this case, there was a 2-week interval between the patient’s initial trauma and subsequent spinal fracture surgery, during which no milky fluid was observed in the thoracic drainage. This timeline, combined with the absence of intraoperative or postoperative evidence of ductal injury, effectively ruled out chylothorax secondary to thoracic or lumbar vertebral fractures. Pedicle screws were placed under O-arm navigation guidance, with intraoperative fluoroscopy and postoperative imaging confirming appropriate screw trajectories without anterior vertebral body breach, making thoracic duct injury highly unlikely.

Subsequent venography revealed that the pleural effusions were caused by malposition of the central venous catheter tip. Two possible scenarios were considered. First, during CVC placement on the day of surgery, the guidewire might have inadvertently injured the vessel wall, allowing the catheter tip to migrate outside the venous lumen. Despite this, fluid administration through the side port proceeded uneventfully, without compromising anesthesia or hemodynamic stability. Second, the catheter may have been appropriately placed initially but displaced during postoperative patient transfers or position changes. In this scenario, infusion through both the main and side lumens could have led to fluid extravasation into the pleural space.

The patient remained under continuous sedation for 4 days post-surgery, and propofol-containing fluids may have leaked into the pleural space during this time. The resulting bilateral pleural effusions impaired respiratory function, delaying ventilator weaning for 3 days. Notably, blood could be aspirated from the side port of the catheter but not from the main port, suggesting that the main lumen had migrated extravascularly, while the side port remained intravascular.

A similar case was reported by Chen Xiaobin’s team,^[5]^ in which a patient developed pleural effusion following internal jugular vein catheterization. The authors attributed the complication to inexperience in needle direction and insertion depth, which likely caused both vascular and pleural injury during repeated attempts. In their case, the catheter was believed to have migrated into the pleural space following a change in patient position from supine to left lateral.

In conclusion, while CVC is generally considered a safe and reliable procedure, it requires a comprehensive understanding of vascular anatomy and adjacent neurovascular structures to minimize the risk of complications. Proficiency in the technical skills, familiarity with potential intraoperative challenges, early recognition of complications, and the implementation of effective preventive measures are essential to reducing adverse events and ensuring the optimal clinical application of this procedure for a broad range of patients.

In this case, vascular perforation occurred during right internal jugular vein catheterization, leading to the unintended diffusion of sedatives into both lungs. This complication resulted in prolonged sedation and delayed awakening, underscoring the potential risks associated with internal jugular vein access. Improper technique may cause vascular injury, alter drug distribution, and ultimately hinder patient recovery.

From this experience, we have identified several important lessons. First, this case reinforces the value of ultrasound guidance as a standard of care for CVC placement to ensure accurate vessel cannulation and mitigate injury risk. Second, heightened vigilance is paramount throughout the procedure. Any acute change in central venous pressure, resistance to infusate flow, or inability to aspirate blood should immediately raise suspicion of catheter malposition or perforation. Finally, obtaining a post-procedural chest radiograph is crucial to confirm proper catheter tip placement and definitively rule out complications like extravasation or pneumothorax, which are essential for patient safety.

## Acknowledgments

We appreciate the patient for providing the informed consent for this study. The authors would also like to acknowledge TopEdit (www.topeditsci.com) for their valuable linguistic support during the revision of this manuscript.

## Author contributions

**Conceptualization:** Zhaolin Xie, Qibiao Zhang.

**Formal analysis:** Zhaolin Xie, Hongyuan Liao.

**Investigation:** Hongyuan Liao.

**Methodology:** Qibiao Zhang.

**Validation:** Qibiao Zhang.

**Writing – original draft:** Zhaolin Xie.

**Writing – review & editing:** Hongyuan Liao, Qibiao Zhang.
